# A case of cerebral tuberculoma mimicking neurocysticercosis

**DOI:** 10.1002/ams2.272

**Published:** 2017-04-04

**Authors:** Hiroko Yuzawa, Yousuke Hirose, Tomonori Kimura, Sho Kimura, Hisanori Sugawara, Asako Yanagisawa, Sono Toi, Takashi Ohashi, Tomohito Sadahiro

**Affiliations:** ^1^ Department of Emergency and Critical Care Medicine Tokyo Women's Medical University Yachiyo Medical Center Yachiyo Chiba Japan; ^2^ Department of Neurology Tokyo Women's Medical University Yachiyo Medical Center Yachiyo Chiba Japan

**Keywords:** Adenosine deaminase, immigrants, interferon gamma release tests, intracranial tuberculoma, neurocysticercosis

## Abstract

**Case:**

A 42‐year‐old Peruvian woman residing in Japan for 11 years with a family history of neurocysticercosis presented to our intensive care unit with fever and intense headache.

Computed tomography indicated multiple micronodular lesions in the brain parenchyma, and cerebral tuberculoma and neurocysticercosis were considered in the differential diagnosis. Neurocysticercosis was initially suspected, and oral praziquantel was initiated. However, because of a high adenosine deaminase level in the cerebrospinal fluid and positive peripheral blood interferon gamma release test result, cerebral tuberculoma was subsequently considered.

**Outcome:**

Antituberculous drugs with steroids were initiated on day 10, after which the symptoms gradually resolved; the patient was discharged on day 29. Gadolinium‐contrast magnetic resonance imaging 8 months later showed reduced nodular shadows, confirming cerebral tuberculoma.

**Conclusion:**

Immediate diagnosis and treatment are imperative for cerebral tuberculoma, a lethal infection. Considering the recent increases in immigration worldwide, increased cases of tuberculoma mimicking neurocysticercosis are expected.

## Introduction

We encountered a case of cerebral tuberculoma associated with intense headache and fever, which mimicked neurocysticercosis. Both diseases are rare in Japan but prevalent in endemic areas such as Latin America, Sub‐Saharan Africa, and Southeast Asia. Recently, immigration from these areas has been increasing. Opportunities to experience similar cases are expected to increase worldwide, and we therefore report this case.

## Case

A 42‐year‐old peruvian woman residing in Japan for 11 years, and who had stayed in Peru for 1 month, 9 years ago, was emergently transferred to our intensive care unit because of generalized tonic seizures. She had suffered from occasional headaches for 3 years and anorexia and weight loss for 4 months. She had presented to a primary care physician 2 weeks ago with exacerbating headache, fever, and night sweats, for which antibiotic therapy was initiated on suspicion of pyelonephritis. She had no previous relevant medical history but had a family history of neurocysticercosis (her older sister in Peru).

On admission, her body temperature was 38.0°C; blood pressure, 95/58 mmHg; heart rate, 108 b.p.m.; respiratory rate, 27 breaths/min; and SpO_2_, 100% (O_2_ 2 L/min). Her consciousness was impaired (Glasgow Coma Scale, E3V3M5), and she was agitated. She had no anisocoria, nuchal rigidity, or hemiplegia.

**Figure 1:**
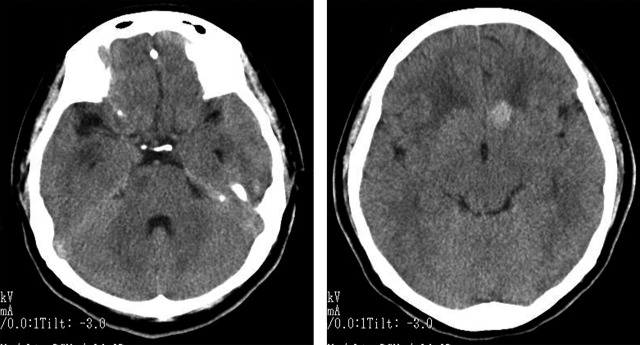
Head computed tomography images on intensive care unit admission of a 42‐year‐old Peruvian woman with fever and severe headaches, showing multiple micronodular lesions in the brain parenchyma.

**Figure 2:**
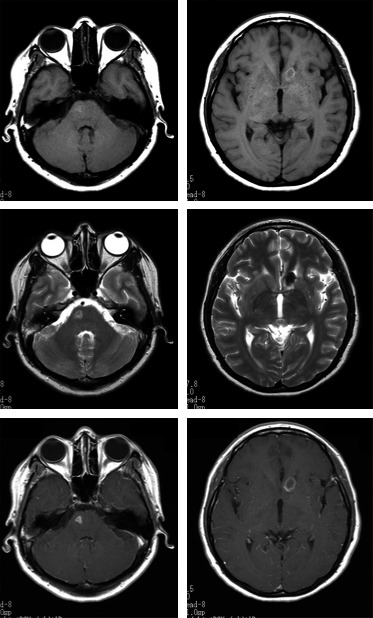
Head magnetic resonance images on intensive care unit admission of a 42‐year‐old Peruvian woman with fever and severe headaches. Upper panels, T1‐weighted images showing an isointense nodular lesion and a hypointense nodular lesion. Middle panels, T2‐weighted images showing the isointense nodular lesion on T1‐weighted image becoming hyperintense, while the hypointense nodular lesion became hypointense with a hyperintense fringe. Lower panels, Gadolinium‐contrasted T1‐weighted images showing ring‐enhancing lesions.

Hematology results indicated no abnormalities; white blood cell count and C‐reactive protein level were within the normal ranges. Chest X‐ray detected no abnormal shadows. Head computed tomography (CT) identified multiple micronodular lesions in the brain parenchyma (Fig. 1). Magnetic resonance imaging (MRI) showed several lesions. Some were isointense on T1‐weighted imaging and hyperintense on T2‐weighted imaging. Others were hypointense on T1‐weighted imaging and showed a hypointense area with a hyperintense fringe on T2‐weighted imaging. Gadolinium‐enhanced MRI showed ring‐enhancing lesions (Fig. 2). There were no findings suggesting bleeding on T2* imaging.

**Figure 3:**
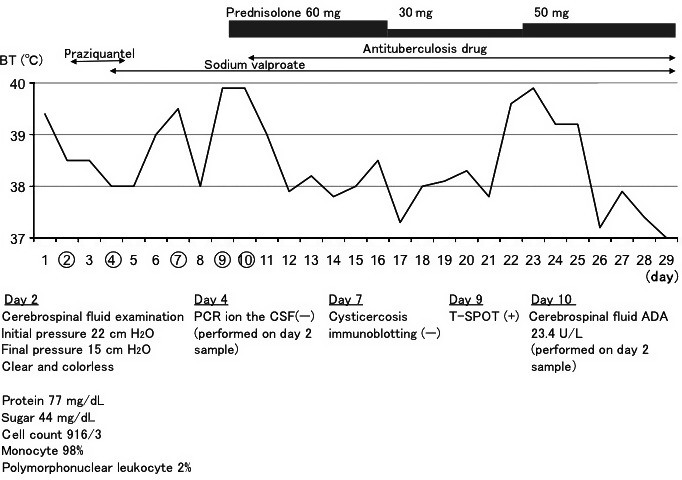
Clinical course of a 42‐year‐old Peruvian woman with cerebral tuberculoma after intensive care unit admission.

After intensive care unit admission, the patient became conscious, but her fever exceeded 39°C and she complained of intense headaches. Antipyretics and analgesics had almost no effect, and she occasionally cried due to severe headaches. Based on the radiologic findings, the patient's home country, and family history, neurocysticercosis was strongly suspected and we started treatment with praziquantel. Although cerebrospinal fluid (CSF) analysis on day 2 suggested tuberculous meningitis (Fig. 3), the CSF findings for tuberculous meningitis are often atypical. Furthermore, CSF polymerase chain reaction (PCR) analysis was negative and no tuberculosis foci were identified at other sites, including the lungs.

Serum immunoblotting assay was negative for *Taenia solium* antigen on day 7; however, on day 9, the peripheral blood interferon gamma release test result (T‐SPOT; Oxford Immunotec, Abingdon, UK) was positive. On day 10, high adenosine deaminase (ADA) activity (23.4 U/L) was detected in the CSF sample collected on day 2. Considering these laboratory findings, cerebral tuberculoma was suspected rather than neurocysticercosis, and we started antituberculous chemotherapy with isoniazid, pyrazinamide, rifampicin, and ethambutol. As the high fever and intense headaches persisted, prednisolone 60 mg/day was started on day 9, which was later adjusted due to exacerbated headaches and persistent fever (Fig. 3). After her symptoms improved and oral food intake was resumed, she was discharged on day 29. The sputum and gastric fluid PCR analyses carried out before the initiation of antituberculosis therapy were negative.

Eight months later, gadolinium‐enhanced brain MRI confirmed reductions in the nodular shadows. Based on her favorable clinical course after the initiation of antituberculous chemotherapy, a diagnosis of cerebral tuberculoma was ultimately established.

## Discussion

Cerebral tuberculoma and neurocysticercosis, which often mimic brain tumors on imaging, are more prevalent in tropical countries than brain tumors.^1^ Although cerebral tuberculoma is usually solitary^2^ and neurocysticercosis often presents as multiple lesions, the differential diagnosis of cerebral tuberculoma with multiple nodular lesions from neurocysticercosis is difficult, owing to similarities in clinical symptoms and CT/MRI imaging findings. Another factor making the differential diagnosis of the two diseases difficult is the partial coincidence of their endemic areas. The fact that the present patient was from Peru is important, as both cerebral tuberculoma and neurocysticercosis are prevalent in this area,^3^ and this initially resulted in an incorrect diagnosis. Considering the extremely high mortality rate of 80% in patients with symptomatic cerebral tuberculoma for ≥2 months,^2^ as in the present case, accurate diagnosis is essential.

In general, biopsy, laboratory data (CSF analysis, immunology), chest radiographs, and family history are considered useful for the differential diagnosis of cerebral tuberculoma and neurocysticercosis.^1^ Herein, biopsy was difficult because the main focus was located in the brain, and chest radiology detected no abnormalities. A family history of neurocysticercosis complicated the differential diagnosis even further.

Additionally, the differential diagnosis of cerebral tuberculoma and neurocysticercosis by diagnostic imaging is difficult, and CT/MRI images were not useful for differentiating the diseases in the present case. The usefulness of magnetic resonance spectroscopy (MRS) has been reported recently. Mukherjee *et al*.^4^ reported that MRS showed a specific lipid peak in cases of tuberculoma, whereas N‐acetylaspartate results in the highest peak in normal brains. Contrastingly, in cases of neurocysticercosis, the highest MRS peak is lactate. Therefore, a high lipid peak on MRS, which is not seen in neurocysticercosis,^4^ is highly specific for tuberculoma in the context of ring‐enhancing lesions.

The keys to the diagnosis in the present case were the CSF ADA levels and peripheral blood T‐SPOT assay. For the diagnosis of central nervous system tuberculosis, the sensitivity and specificity of ADA levels in the CSF (>5.8 U/L) are reportedly 89% and 73%, respectively, and those for the T‐SPOT assay are 71% and 57%, respectively.^5^ For the diagnosis of neurocysticercosis, the sensitivity and specificity of antibody testing are 70–90% and nearly 100%, respectively, and the sensitivity of antigen testing is also high.^3^ Unfortunately, the result of the antigen testing in the present case was not reported until several days after sample submission.

The patient had a persistent fever with headaches intense enough to make her cry and for which analgesics were ineffective. However, concomitant administration of therapeutic drugs for the two suspected diseases makes therapeutic diagnosis impossible. Herein, we first chose to administer neurocysticercosis treatment, owing to the patient's family history; this delayed the start of antituberculous chemotherapy. According to the guideline for taeniasis and cysticercosis, fever is one symptom suggesting other diagnoses.^6^ Therefore, we needed to consider other diseases than neurocysticercosis. Although the CSF PCR for tuberculous meningitis was negative, its sensitivity is only 56%,^7^ and a diagnosis of cerebral tuberculoma could not be excluded. In retrospect, despite antituberculosis drugs being associated with a high risk of adverse effects, antituberculous chemotherapy should be started before the definitive diagnosis, because tuberculoma is a fatal disease.

## Conclusion

Differential diagnosis of cerebral tuberculoma and neurocysticercosis is difficult because of similarities in the clinical symptoms and routine imaging data. A high CSF ADA level and positive T‐SPOT assay were helpful in the differential diagnosis in the present case. As cerebral tuberculoma is a fatal disease, immediate diagnosis and early initiation of treatment are imperative.

## Disclosure

Based on the ethical consideration in the author guidelines, the patient has provided permission to publish these features of her case on the condition that patient identity remains undisclosed.

## Conflict of interest

None declared.

## References

[1] Gothi R . Consider tuberculoma and cysticercosis in the differential diagnosis of brain tumour in tropical countries. BMJ 2013; 347: f6604. 24196502 10.1136/bmj.f6604

[2] Oncul O , Baylan O , Mutlu H , Cavuslu S , Doganci L . Tuberculous meningitis with multiple intracranial tuberculomas mimicking neurocysticercosis clinical and radiological findings. Jpn. J. Infect. Dis. 2005; 58: 387–9. 16377875

[3] Nash TE , Garcia HH . Diagnosis and treatment of neurocysticercosis. Nat. Rev. Neurol. 2011; 7: 584–94. 21912406 10.1038/nrneurol.2011.135 PMC3350327

[4] Mukherjee S , Das R , Begum S . Tuberculoma of the brain‐A diagnostic dilemma: magnetic resonance spectroscopy a new ray of hope. J. Assoc. Chest Physicians 2015; 3: 3–8.

[5] Kim SH , Cho OH , Park SJ *et al* Rapid diagnosis of tuberculous meningitis by T cell‐based assays on peripheral blood and cerebrospinal fluid mononuclear cells. Clin. Infect. Dis. 2010; 50: 1349–58. 20380567 10.1086/652142

[6] Nash TE , Garcia HH , Rajshekhar V , Del Brutto OH . Chapter 2: Clinical cysticercosis: diagnosis and treatment In: Murrell KD (ed.). WHO/FAO/OIE Guidelines for the Surveillance, Prevention and Control of Taeniasis/Cysticercosis. Paris: OIE, 2005; 19.

[7] Pai M , Flores LL , Pai N , Hubbard A , Riley LW , Cloford JM Jr . Diagnostic accuracy of nucleic acid amplification tests for tuberculous meningitis: a systematic review and meta‐analysis. Lancet Infect. Dis. 2003; 3: 633–43. 14522262 10.1016/s1473-3099(03)00772-2

